# Comparative Performance of Ante-Mortem Diagnostic Assays for the Identification of *Mycobacterium bovis*-Infected Domestic Dogs (*Canis lupus familiaris*)

**DOI:** 10.3390/pathogens14010028

**Published:** 2025-01-03

**Authors:** Conor O’Halloran, Paul Burr, Danielle A. Gunn-Moore, Jayne C. Hope

**Affiliations:** 1 The Roslin Institute and Royal (Dick) School of Veterinary Studies, University of Edinburgh, Midlothian EH25 9RG, UK; danielle.gunn-moore@ed.ac.uk (D.A.G.-M.); jayne.hope@roslin.ed.ac.uk (J.C.H.); 2 Biobest Laboratories, Edinburgh EH26 0BE, UK; paul.burr@biobest.co.uk

**Keywords:** canine, diagnosis, dog, tuberculosis, mycobacterium, IGRA, serology

## Abstract

The domestic dog (*Canis lupus familiaris*) is a competent host for *Mycobacterium* (*M.*) *bovis* infection but no ante mortem diagnostic tests have been fully validated for this species. The aim of this study was to compare the performance of ante mortem diagnostic tests across samples collected from dogs considered to be at a high or low risk of sub-clinical *M. bovis* infection. We previously tested a total of 164 dogs at a high risk of infection and here test 42 dogs at a low risk of infection and 77 presumed uninfected dogs with a combination of cell-based and/or serological diagnostic assays previously described for use in non-canid species. The interferon-gamma release assay (IGRA) using peripheral blood mononuclear cells (PBMCs) identified the highest number of test-positive animals (85, 52%), with a suggested specificity of 97.3%, whilst a whole-blood IGRA was found to be unreliable. The production of antigen-specific tumour necrosis factor-alpha (TNF-α) by PBMC in response to a cocktail of ESAT-6 and CFP-10 peptides correlated very strongly with the overall IGRA results, suggesting future diagnostic potential. All three serological assays employed in this study (Idexx *M. bovis* Ab ELISA, [Idexx Laboratories, Westbrook, ME, USA], DPP VetTB lateral flow assay [Chembio, Medford, NY, USA], and comparative PPD ELISA [in-house]) identified seropositive dogs but, overall, the test-positive rate for the serological assays was only one third that of the cellular-based assays. Circulating serum cytokine concentrations of interferon gamma and TNF-α were not statistically different between the high- and low-risk groups of dogs. While many dogs in the high-risk group had serum biochemical abnormalities, these did not correlate with the findings from the diagnostic TB tests. This study demonstrates, for the first time, the utility of two cellular and three serological assays for detecting sub-clinical *M. bovis* infections of dogs. Whilst the data suggest a high test specificity for all assays evaluated, further work is needed to validate the sensitivity and specificity of individual or combinations of tests using sufficient numbers of dogs of a known infection status.

## 1. Introduction

The domestic dog (*Canis lupus familiaris*) is, like many mammalian species, a competent host for *Mycobacterium* (*M.*) *bovis* infection [1,2,3]. Although clinical cases of tuberculosis (TB) due to infection with *M. bovis,* or other members of the *M. tuberculosis* complex (MTBC), are considered rare in dogs and generally only occur sporadically [4], we previously investigated a fulminant disease outbreak caused by *M. bovis* infection within a large group of kennel-housed working foxhounds in the UK [5]. The investigation of this outbreak was impeded in its speed and by the lack of available ante mortem diagnostic tests for identifying *M. bovis* infections in domestic dogs [1,3]. The tests employed therefore had to be adapted and interpreted in real time, based on the extrapolation of performance data from non-canine species.

Interferon gamma (IFN-γ) release assay (IGRA) tests have been developed on the principle of quantitatively evaluating antigen-specific IFN-γ production by peripheral circulating effector memory T-cells following in vitro stimulation [6,7,8]. The first IGRA was designed to increase the sensitivity of *M. bovis* TB testing in cattle [9], and the resulting BOVIGAM^®^ assay holds World Organisation for Animal Health (WOAH) validation, with a reported in-field sensitivity in GB of 90% [8] and specificity of 96.5% [10]. IGRA tests have subsequently been adapted to identify active and latent TB in human patients with at least equivalent sensitivity and increased specificity when compared to the intradermal tuberculin skin test (TST) [11,12,13,14]. Similarly, IGRA test protocols have been developed for use in domestic cats with a high sensitivity and specificity for the detection of MTBC infections [15] and for the detection of *M. tuberculosis* infections in dogs—while the same study found the intradermal TST to be unreliable in dogs [16].

The IGRA used in this study was specifically developed to test dogs for subclinical *M. bovis* infection, and was based on the protocol used for testing domestic cats (Mitchell et al. 2021) [15]; the antigens used were purified protein derivative (PPD) from *M. avium* (PPDA), PPD from *M. bovis* (PPDB), and a cocktail of peptides derived from the immunodominant proteins 6 kDa early-secreted antigenic target (ESAT-6) and 10 kDa culture filtrate protein (CFP-10). PPDA is frequently used with PPDB as a comparator in both IGRA and TST tests to assess and mitigate for exposure/sensitisation to, or infection with, environmental mycobacteria species. Infection with a MTBC mycobacteria is confirmed if the IFN-γ response of an animal is greater to PPDB than to PPDA [9,17].

The proteins ESAT-6 and CFP-10 are both encoded on the RD-1 region of most MTBCs, including *M. bovis*, and a small number of non-tuberculous mycobacteria (NTM) [18]. They are secreted proteins that form a heterodimeric complex in a 1:1 ratio, which has been consistently associated with phagocyte lysis and pathogen virulence [18]. The presence of a concurrent response to the synthetic peptide combination of ESAT-6/CFP-10, as well as PPDB, indicates infection with an RD-1^+^ MTBC mycobacteria (i.e., it excludes infection with *M. microti* or a previous vaccination with *M. bovis*-BCG) [18,19].

A number of studies have tried to improve the sensitivity and/or specificity of IGRA tests. The overall performance of an IGRA, when used to test cattle for *M. bovis* or humans for *M. tuberculosis* infection, can be increased by the measurement of additional cytokines secreted by antigen-specific cells within the blood [20]. This includes tumour necrosis factor-alpha (TNF-α), which has a well-described role in the mammalian host immune response to mycobacteria [20,21].

The bovine (BOVIGAM^®^) and human (QuantiFERON-TB^®^ Gold) IGRA assays are both performed on whole blood without first needing to isolate peripheral blood mononuclear cells (PBMCs), as is required for the IGRA testing of cats, camelids, and the human T-SPOT.*TB*^®^ test [21,22,23,24]. The use of whole blood rather than PBMC makes the former assays quicker and cheaper to conduct and so would be an ideal development for canine TB testing.

IGRA and TST assays evaluate the cell-mediated adaptive immune response (CMI) of the subject, which dominates the reaction to intracellular pathogens, including mycobacteria [25]. However, as above, skin test responses in dogs have been shown to be unreliable [16]. Diagnostic serological assays, while generally recognised as having lower sensitivity than CMI assays, have been used both experimentally and commercially for the diagnosis of MTBC infections in a number of species [26,27,28,29,30,31,32]. It is recognised that combining serological and CMI diagnostic tests may provide added value by increasing infection detection; e.g., Bezos et al. [32] showed an increase in the overall diagnostic sensitivity for both cattle and goats with MTBC infections.

*M. bovis* is an endemic pathogen of major significance to the UK farming industry as the causative agent of bovine tuberculosis (bTB) [10]. In England, the counties with the highest prevalence of *M. bovis*, as defined by the incidence of bovine infections, comprise the high-risk area (HRA), and lower but still significant disease prevalence occurs within counties of the edge area (EA). Control measures implemented for cattle infections amount to an estimated cost of £100 million annually, and while the latest government statistics show a decrease in herd incidence across GB, the number of cattle slaughtered due to bovine TB remains high and eradication remains challenging [33].

Non-bovine incidents are increasingly being investigated for their potential role in the epidemiology of the disease, either as possible reservoirs of infection or as sentinel species, but species-specific diagnostic tests are often lacking, which impedes such investigations [34,35]—in the case of canids this was highlighted by the fulminant outbreak of *M. bovis* infection in a pack of foxhounds [5].

The aim of this study was to compare available diagnostic tests and assays to samples collected from individual dogs considered to be at high, low, or no risk of *M. bovis* infection, to gauge their potential usefulness going forward.

## 2. Materials and Methods

### 2.1. Study Dog Samples

Diagnostic blood samples from animals considered to be at a high risk of sub-clinical infection with *M. bovis* (high-risk group, HRG) were those taken from 164 kennel-housed foxhounds during an outbreak of TB due to *M. bovis* that occurred between December 2016 and July 2017 (Table 1) [5]. Whole-blood and PBMC stimulation assays were conducted at the time of sampling, whilst serological assays were performed subsequently on aliquots of serum separated and frozen at −80 °C within 24 h of collection. We also tested a total of 119 opportunistically collected blood samples (i.e., remnant blood from samples taken for a clinical reason unrelated to this project); 77 from presumed TB-free dogs (TB-free group), and 42 dogs with some degree of potential exposure to cats or dogs with a mycobacterial infection (low-risk group, LRG).

The LRG comprised 45 serum samples and 74 PBMC samples (there were no LRG dogs where both serum and PBMCs were available due to the remnant nature of sample acquisition). Remnant samples were only included in this study if, prior to blood sample collection, the dog had not been treated with immunomodulatory medications, e.g., non-steroidal anti-inflammatories (NSAIDs), chemotherapeutic agents, or corticosteroids, within 14 days prior to sample collection. Dogs were excluded if they were pre-treated with antibiotics with efficacy against mycobacteria, including fluoroquinolones, macrolides/azides, or doxycycline, within the same 14-day period. Dogs were not excluded if they had been treated with antimicrobial agents if these would be ineffective against mycobacteria, such as a penicillin or cephalosporin. Similarly, dogs were not excluded if they had been treated with non-immunomodulatory analgesic medications, e.g., opioids.

This study was conducted following approval from the School of Veterinary Medicine Ethical Review Committee at the University of Edinburgh; all relevant guidelines and regulations were adhered to throughout.

### 2.2. IGRA Using Isolated PBMC

IGRA assays for the 164 dogs in the HRG were conducted as previously reported in [5]. The same assay was used to test the PBMC isolates from 74 dogs in the LRG (PBMC samples, Table 1). The basic assay protocol has been validated and previously published for use in domestic cats [15]. A sample of up to 5 mL heparinised whole blood was taken from each animal and transported to the laboratory at ambient temperature within 18 h. Upon receipt, blood was diluted 1:1 with Hanks Balanced Salt Solution (HBSS, Gibco, London, UK) and layered over Histopaque 1077 (Sigma, Gillingham, UK) before centrifugation at 800× *g* for 40 min at room temperature. PBMCs were removed from the interface, washed with HBSS, and re-suspended in complete culture media (RPMI 1640 containing 100 µg/mL L-glutamine, 10% foetal bovine serum, 100 µg/mL penicillin, 100 U/mL streptomycin, 5 × 10^−5^ M 2-mercaptoethanol, and non-essential amino acids) to 2 × 10^6^/mL. A total of 100 μL of PBMC suspension were stimulated in duplicate with PPDA or PPDB (Lelystad, Prionics, The Netherlands) both at a final dilution of 1:100 as well as a peptide cocktail of ESAT-6/CFP-10 at a final concentration of 5 μg/mL (Lionex, Germany), a mitogen-positive control of phorbol myristate acetate plus calcium ionophore (PMA/Ca, Sigma, UK, 50 ng/mL and 1 µg/mL respectively), and finally a culture medium only, i.e., an unstimulated negative control was included for each animal.

Cells were incubated for four days at 37 °C/5% CO_2_, after which the supernatants were removed, and duplicates pooled, for quantification of IFN-γ by ELISA. Supernatants were either directly assayed or stored at −80 °C until required. The IFN-γ ELISA was conducted using a commercially available canine-specific ELISA kit (DY781B, R&D Systems, Europe Ltd., Abingdon, UK) according to the manufacturer’s instructions.

Supernatant from each cell culture condition was assayed in duplicate. Optical density (OD) values were measured at wavelengths of 450 nm and 630 nm; the replicate OD (450–630 nm) values for each condition were averaged to give the final OD values, and standard deviations were calculated. Where replicates differed by more than 30% from the mean, the test was considered invalid.

During the foxhound outbreak, IGRA test cut-offs had been prospectively set to identify positive dogs [5]. It was determined that a statistically significant response to any of the three test antigens (PPDB, PPDA, or ESAT6/CFP10) above the negative condition response was indicative of a biologically significant T-cell response. The responsiveness threshold for each dog was defined as the mean OD value of the sample media negative control plus two standard deviations (2SD). The mean antigen-specific response minus 2SD must exceed this for a positive test (i.e., non-overlapping 2SD). Similarly a PPDB-biased response was one where the mean PPDB response minus 2SD was greater than the mean PPDA response plus 2SD. For the test to be considered valid, the sample-positive control response must also be positive by the same criteria when compared to the negative sample control. As many of the PBMC positive control and antigen-specific response values were above the linear range of the test kit standard curve (recombinant IFN-γ), the OD values, rather than IFN-γ concentration, were used for test result interpretation.

### 2.3. TNF-α Assay Using PBMC

TNF-α was measured in stimulated (with media-only, PPDA, PPDB, or ESAT-6/CFP10 and PMA/CA as above) cell supernatants from a randomly selected subset of 45 dogs from the HRG by commercial ELISA (DY1507, R&D Systems, Europe Ltd., UK) according to the manufacturer’s instructions. Since PMA/Ca stimulation did not work well as a TNF-a positive control (i.e., it did not induce TNF-α in any dog sample tested, even where antigen-specific TNF-α responses were observed), concanavalin A (ConA) was used as a positive control mitogen in one supporting experiment using 3 healthy dogs (from the blood donor subset, Table 1). PBMCs were isolated and incubated in the same conditions as described above but with the addition of ConA at a final concentration of 25 µg/mL. After incubation for four days at 37 °C/5% CO_2_, the culture supernatant was assayed for TNF-α by ELISA. Samples were interpreted as positive where the mean antigen-specific response minus 2SD was greater than the mean media sample-negative control plus 2SD. Similarly, a PPDB-biased response was one where the mean PPDB response minus 2SD was greater than the mean PPDA response plus 2SD.

### 2.4. IGRA Using Whole Blood

To compare the diagnostic usefulness of whole blood versus PBMC stimulation methods, a whole-blood assay was conducted on the same subset of 45 dogs in the HRG tested in Section 2.3. Whole-blood stimulation was performed using heparinised whole blood. One millilitre of heparinised whole blood was added to each of five separate wells of a 12-well tissue culture plate (ThermoFisher Scientific, Waltham, MA, USA) to which the following had been added: (a) 25 µL phosphate-buffered saline (PBS, pH 7.2); (b) 25 µL PMA/Ca (Sigma, UK) to final concentrations of 50 ng/mL and 1 µg/mL, respectively; (c) PPDA or PPDB, to a final dilution of 1:100 (Lelystad, Prionics, Netherlands); (d) 25 µL of purified ESAT-6 to a final concentration of 5 µg/mL; and (e) 25 µL of combined ESAT-6/CFP-10, to a final concentration of 5 µg/mL each. The plates were incubated overnight (approximately 16 h) at 37 °C/5% CO_2_. The following day, the plates were centrifuged at 800× *g* for 15 min and the plasma was removed from the cellular fraction before being stored frozen at −80 °C. The concentration of IFN-γ in the samples was subsequently determined by ELISA (DY781B, R&D Systems, Europe Ltd., UK). Plasma samples from each condition were assayed in duplicate. The mean replicate OD (450–630 nm) values were used to determine the concentration of IFN-γ against a recombinant canine IFN-γ standard curve (R&D Systems) and values are reported in pg/mL.

### 2.5. Serum Antibody Testing

**DPP VetTB lateral flow assay:** Sera from each of the 164 HRG dogs and the 45 sera from TB-free dogs (Table 1) were tested using the DPP VetTB lateral flow test (Chembio Diagnostic Systems, Inc., Medfords, NY, USA) as previously reported (O’Halloran et al. 2018). Briefly, 30 μL of serum was applied to a single cassette and washed across two antigen lines (MPB83 and ESAT-6/CFP-10) with the kit buffer. After 5 min, a further buffer application was applied to wash the colloidal gold-detecting reagent across the antigen and test control lines. After 15 min, each cassette was visually inspected for QC purposes (a visible positive control line) and the antigen-specific antibody binding was quantified by inserting the cassette into an optical reader (Optricon DPP Reader, Chembio) that measures the reflectance of the response produced to both antigen lines individually as relative light units (RLUs).

**IDEXX M. bovis ELISA:** Sera from the same animals (HRG and TB-free) were also tested using the cattle Idexx *M. bovis* antibody (Ab) ELISA kit (Idexx Laboratories, Inc., Westbrook, ME, USA), performed with minor modifications to detect canine antibodies. The kit secondary anti-bovine detection antibody was retained for the kit-ELISA plate positive and negative bovine controls only, while antigen-bound canine antibodies were detected using protein-G conjugated to horseradish peroxidase (HRP) (which binds the Fc region of antibodies of numerous species). Serum samples were diluted 1:50 and added to the ELISA plate in duplicate, together with plate positive and negative controls, and ELISA plates were incubated for one hour at room temperature. Plates were washed with wash buffer (supplied with the kit), and then incubated with the secondary detection reagents (protein-G-HRP for canine samples and kit anti-bovine reagent for plate controls) for 30 min at room temperature and then washed again. Substrate (supplied with the kit) was added to each well (100 µL/well), the plates were developed for 15 min at room temperature, and the reaction was stopped by adding 100 µL/well of stop buffer (supplied with kit). Absorbance values were read at 450 nm according to the manufacturer’s instructions and the mean of each sample replicate calculated as a final OD value test result.

The HRG and TB-free group results were compared using a Mann–Whitney U test with a *p*-value of less than 0.05 considered to be significant.

**Comparative PPD ELISA:** A comparative antibody ELISA was conducted using a method similar to that used previously for cats (Mitchell et al., 2023) [36]. ELISA test plates (NUNC Maxisorp Immmunoplate F96, Scientific Laboratory Supplies Ltd., Nottingham, UK) were coated with 100 µL of test antigen (PPDA or PPDB) diluted to 1:100 in carbonate–bicarbonate buffer (pH 9.6, Sigma Aldrich, UK). Plates were sealed and incubated at room temperature (RT) overnight. Plates were washed with PBS containing 0.05% Tween 20 and were blocked for one hour at RT with blocking buffer comprised of 4% bovine serum albumin (BSA; Sigma Aldrich, UK) in PBS. Serum from each dog (HRG, *n* = 163 and LRG, *n* = 45, Table 1) was diluted 1:50 in 2% BSA in PBS. After the plates were washed, 50 µL of diluted serum was added to test wells in duplicate, and blocking buffer was added to two wells on each plate (blank control). Plates were sealed and incubated at RT for two hours. Plates were washed again and 50 µL of detection antibody (HRP-conjugated goat-anti-canine IgG; Euroimmun, Lübeck, Germany) was added to each well and incubated for 45 min at RT. Following incubation, plates were again washed and 50 µL of 3,3′,5,5′-tetramethylbenzidine (TMB; Sigma Aldrich,) was added. After a 15 min incubation, 25 µL of 2M H_2_SO_4_ stop solution was added.

Absorbance was read at 450 nm and 630 nm, test results for each dog were calculated as the mean OD (450–630 nm) of replicate wells with the mean OD (450–630 nm) of blank control wells from that plate subtracted. For the comparative test, the final OD value for PPDA was subtracted from the value for PPDB, giving an overall ΔPPD OD value. Results were compared using a Mann–Whitney U test with a *p*-value of less than 0.05 considered to be significant.

### 2.6. Serum IFN-γ and TNF-α ELISA

Neat serum from sampled dogs (HRG, *n* = 163 and LRG, *n* = 45) was assayed for IFN-γ and TNF-α using commercial ELISA kits as described above (Section 2.2 and Section 2.3); 50 µL of serum was assayed in duplicate for each dog. The calculated OD value (mean of the replicate OD (450–630 nm) values) was used to determine the concentration of IFN-γ or TNF-α, respectively, against the standard curves. Values were calculated in pg/mL; the results were statistically compared using a Mann–Whitney U test with a *p*-value of less than 0.05 considered to be significant.

### 2.7. Haematology and Serum Biochemistry

Routine haematology and serum biochemistry profiles were performed on half of the HRG (*n* = 82) selected at random at the Easter Bush Pathology Unit (University of Edinburgh).

## 3. Results

### 3.1. IGRA and TNF-α PBMC Stimulation Assays

**Figure 1:**
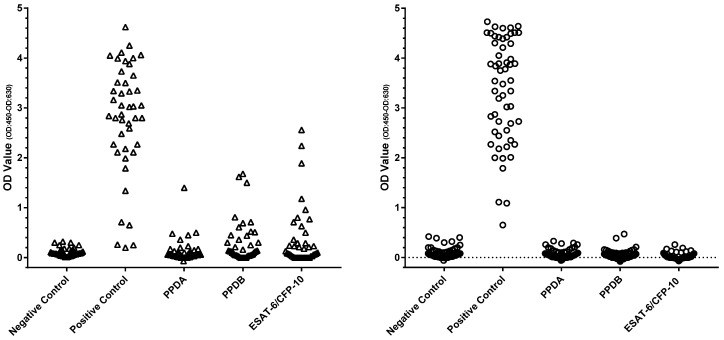
Legend: Results of the PBMC IGRA test for 164 HRG (Δ) and 74 LRG TB-free group (○) dogs. Each symbol shows the mean OD value of duplicate IFN-γ ELISA absorbance measurements for an individual dog.

In total, 164 dogs at a high risk of subclinical *M. bovis* infection were tested by PBMC IGRA, and the results are shown in Figure 1. Only 11 tests failed and required repetition to provide a viable result; the most frequent reason for this (*n* = 6) was that there was a statistically insignificant response to the PMA/Ca-positive control; in some cases, there was clotting of the blood sample, which precluded the isolation of PBMC (*n* = 4), and one test required repetition due to poor replicate ELISA values.

Of the 164 high-risk dogs, 85 (52.0%) showed positive responses to either PPDA, PPDB, or ESAT-6/CFP-10). Seventy-seven (90.1%) of these eighty-five dogs displayed a significant response bias to PPDB over the PPDA response, i.e., 47% of all HRG dogs tested displayed a response pattern indicative of MTBC infection. Within this group of 77 dogs, 48 (62.3%) also responded to the ESAT-6/CFP-10 peptide cocktail, i.e., a response pattern highly suggestive of *M. bovis* infection. In addition, five test-positive individuals responded to ESAT-6/CFP-10 and no other test antigens whilst another three responded only to PPDA.

The IGRA results for the 74 LRG and TB-free dogs are shown in Figure 1. Among these dogs, 14 (19.4%) showed positive responses to either PPDA, PPDB, or ESAT-6/CFP-10). However, in contrast to the HRG, 12 (85.7% of these 14) of these responses were biased toward PPDA compared to PPDB, which is suggestive of exposure to/infection with environmental mycobacteria rather than MTBC species. Only two animals in this group showed a positive bias toward PPDB above PPDA, which is indicative of MTBC infection, suggesting a test specificity of 97.3%. Neither of these dogs responded to the ESAT-6/CFP-10 peptides. Retrospective investigation identified that these two dogs had been previously cohabitant with the HRG and/or been raw-fed. Were these dogs to be removed as dangerous contacts, the test specificity would therefore be higher at ~100%.

**Figure 2:**
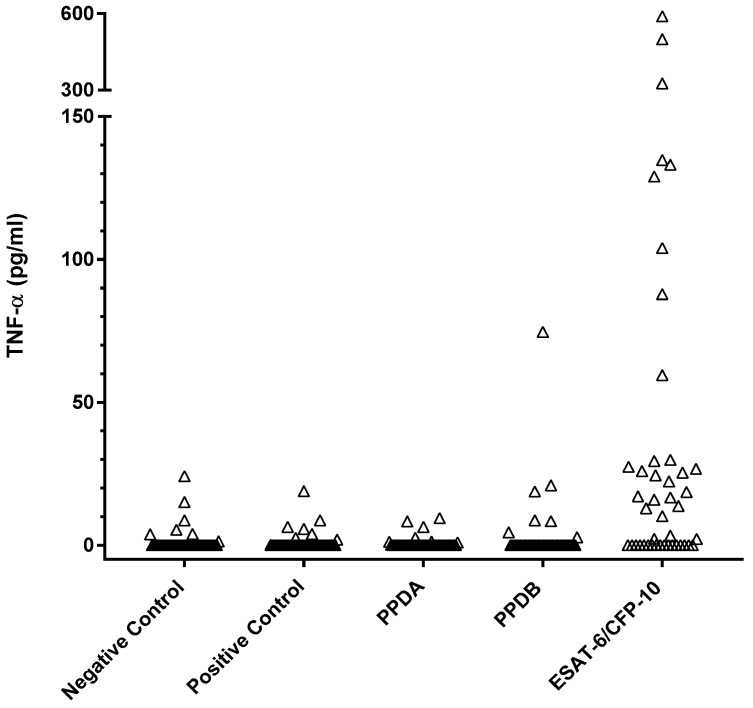
Legend: Results of TNF-α ELISA conducted on the same PBMC supernatant as the IGRA tests in a randomly assigned sub-group of 45 high-risk group dogs. Each symbol shows the mean OD value of duplicate TNF-α ELISA absorbance measurements for an individual dog.

PBMC culture supernatants from 45 of the HRG dogs were assessed for antigen-specific TNF-α (Figure 2). Three of the forty-five dogs had statistically significant quantities of TNF-α in the supernatant of cells stimulated with PPDB compared to the negative control condition. The same three animals, along with an additional twenty-five individuals, were found to have significant levels of TNF-α in the supernatant of PBMC stimulated with the ESAT-6/CFP-10 peptide cocktail.

Comparing the concentration of ESAT6/CFP10-specific TNF-α production with the overall IGRA positivity (i.e., either a PPDB bias or a positive ESAT-6/CFP-10 response) showed that these two tests, interpreted in parallel, identified 29 (64.4%) test-positive and 16 test-negative individuals. These tests showed very strong agreement (Cohen’s kappa coefficient, κ = 0.83).

**Figure 3:**
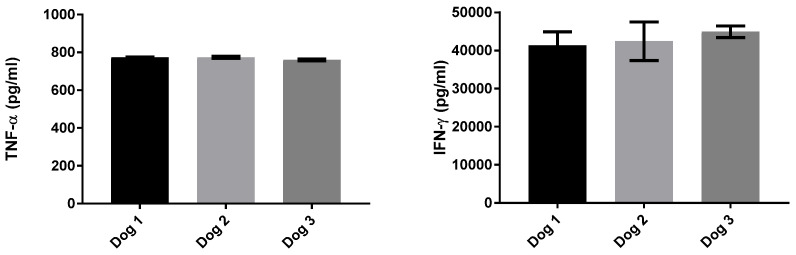
Mitogen-induced TNF-α and IFN-γ. Legend: Concentration (replicate mean and standard deviation) of TNF-α and IFN-γ produced by PBMC stimulated for four days at 37 °C/5% CO_2_ with PMA/Ca/ConA. The addition of Con A to the mitogen mix induced TNF-α in all 3 dogs, whereas all previous PBMC incubations with PMACa did not induce detectable TNF-α.

However, the mitogen PMA/Ca used as a sample-positive control for the IGRA test did not stimulate canine TNF-α production. As we could not re-run the HRG dog stimulations to include an additional mitogen, we compared the IFN-γ and TNF-α production from three healthy dog PBMC samples stimulated with a cocktail of PMA/Ca and concanavalin A (ConA). In this instance, TNF-α and IFN-γ were both produced (Figure 3). Therefore, it was felt that the lack of a TNF-α response to the PMA/Ca-positive control in the HRG group did not invalidate the positive antigen-specific responses observed to PPDB and ESAT-6/CFP10; rather, it indicates that the positive control was not optimal for canine samples and that future assays to measure this and potentially other cytokines should consider various mitogen options for sample-positive controls.

**Figure 4:**
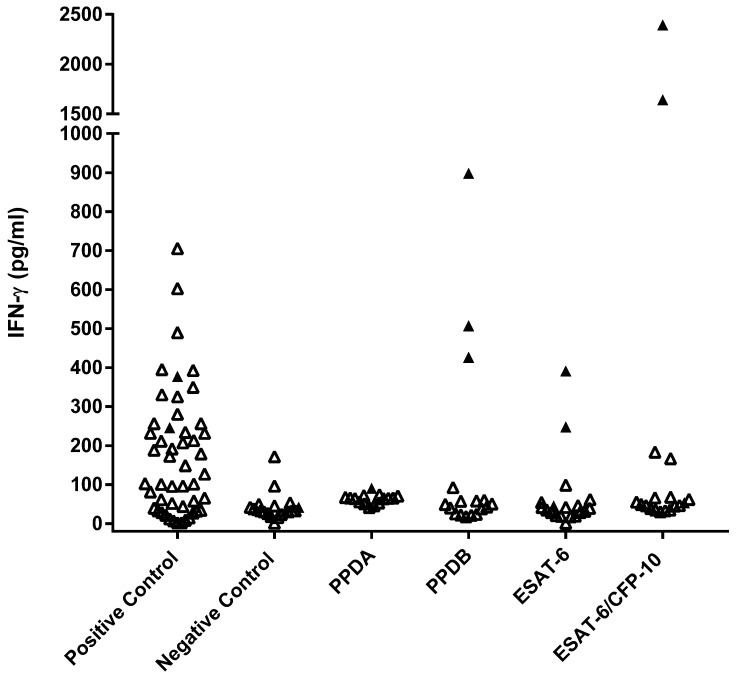
Legend: Results of the whole-blood IGRA test for a randomly assigned sub-group of 45 of the high-risk group of 164 dogs. Concentrations were calculated using the mean of duplicate IFNγ ELISA absorbance measurements against the standard curve. Filled triangles show a positive test response to EC peptide or ESAT-6 or where PPDB > PPDA.

The same subset of 45 HRG dogs was tested using a whole-blood IGRA (Figure 4). Overall responses were low, with three individuals showing responses indicative of MTBC infection (PPDB > PPDA), two of which also showed responses to the peptide cocktail and ESAT-6 protein. Comparing these results with those from the PBMC IGRA showed poor agreement between the two tests (κ = 0.20).

### 3.2. Serum Antibody Assays

**Figure 5:**
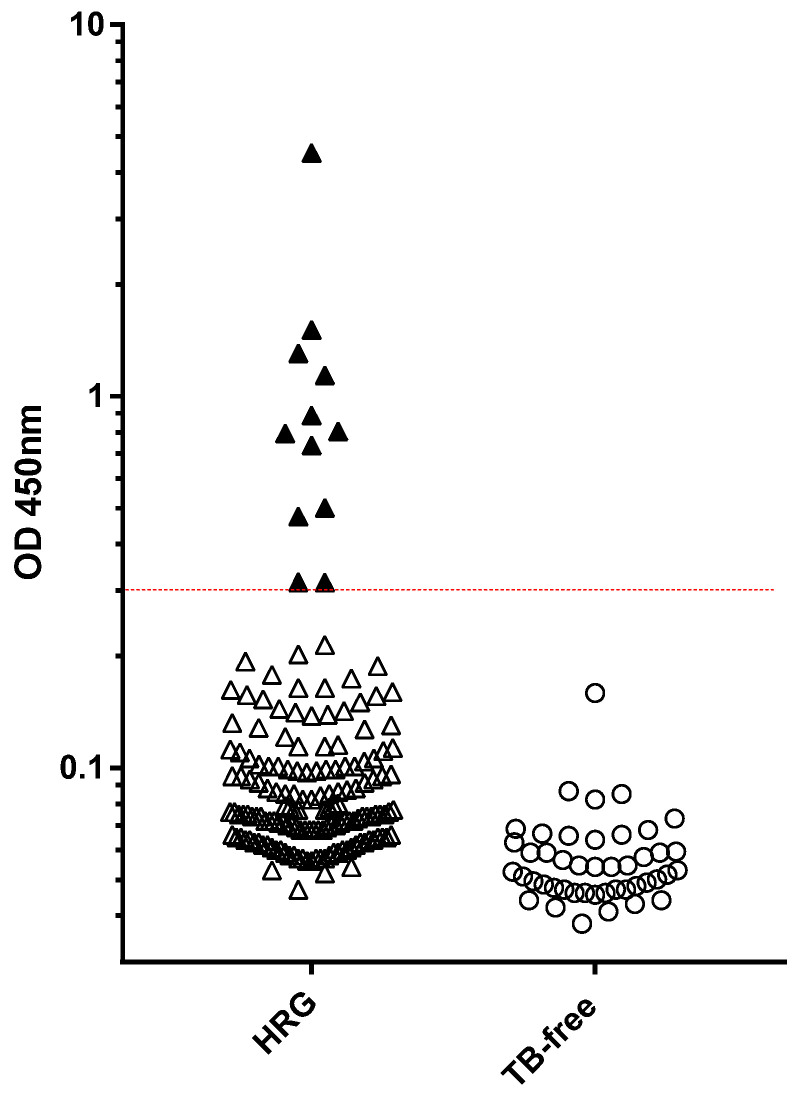
Legend: Serology results for dogs in the high-risk group (HRG, *n* = 163) and TB-free group (*n* = 45) obtained using the Idexx *M. bovis* Ab test. The results shown are the mean OD values of duplicate measurements for individual dog samples at 450 nm absorbance. The test-positive cut-off value of OD 0.3 is shown by a horizontal line. Solid symbols show test-positive samples.

The **Idexx *M. bovis* Ab ELISA** (Figure 5) showed a statistically significant difference between the OD values for the HRG (*n* = 163) and TB-free (*n* = 45) dog groups (Mann–Whitney U = 880.5, *p* < 0.001). In deciding upon a preliminary test cut-off for the canine Idexx assay, we took into account both the cattle Idexx test protocol and the modified Idexx test as applied to camelid TB testing at APHA, both of which approximate to an OD cut-off value of ~0.3 to provide tests of high specificity. The application of a cut-off value of 0.3 to the canine Idexx test data resulted in 100% test specificity for this TB-free group, and identified 12 test-positive dogs within the HRG (7.4%).

**Figure 6:**
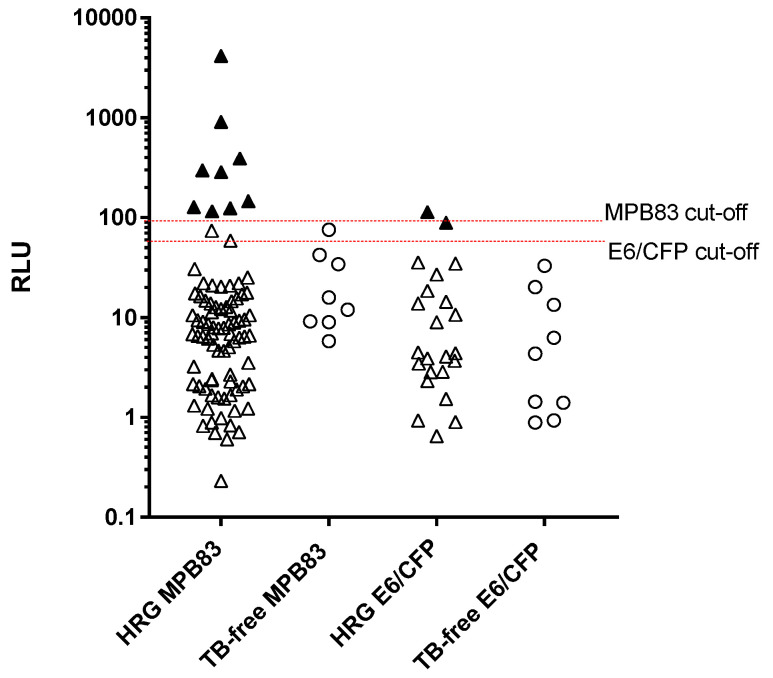
Legend: Serology results for dogs in the high-risk group (HRG, *n* = 163) and TB-free group (*n* = 45) obtained using the DPP VetTB assay. Triangles represent individuals from the HRG and circles represent individuals from the TB-free group. Results are given as Optricon relative light units (RLU), and one symbol represents one dog sample. The test-positive cut-off values for each antigen are shown by the horizontal lines at 95 RLU for MPB83 and 60 RLU for ESAT-6/CFP-10 (E6/CFP). Solid symbols indicate positive samples. Since the axis is logarithmic, only values greater than zero are plotted. For this graph, 291 values overall were zero or negative, so are not included on the graph.

The **DPP VetTB assay** (Figure 6) showed significantly higher RLU values for the HRG compared to the TB-free dogs (Mann–Whitney U = 2731, *p* = 0.003). In deciding upon preliminary cut-offs for the MPB83 and ESAT6/CFP-10 antigens in this test, a preliminary ROC analysis for the MPB83 RLU values suggested a cut-off of >95 to provide a specificity of 100%. A similar analysis for ESAT-6/CFP-10 was not viable due to the very low number (*n* = 2) of sero-positives; however, there was a clear divide between these two sero-positives and test-negatives, of between 40 and 80 RLU, and therefore >60 RLU was chosen as the cut-off for the ESAT-/CFP-10 antigen line for the purpose of this study. Applying these cut-offs to the study cohorts provided a 100% specificity, and identified 11 HRG dogs (6.7%) as test-positive; 9 of these were positive to MPB83 only, and 2 were positive to ESAT-6/CFP-10 only.

**Figure 7:**
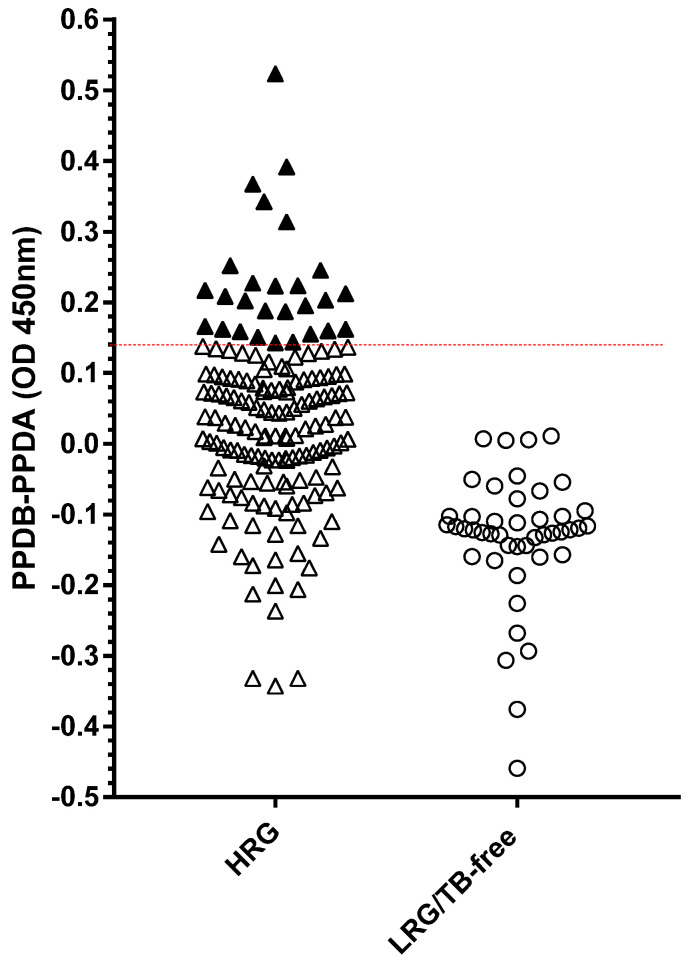
Legend: Serology results for dogs in both the high-risk group (HRG, *n* = 163, triangles) and low-risk group (LRG/TB-free *n* = 45, circles) obtained using a comparative PPD antibody ELISA. The results shown are mean values of duplicate measurements calculated by subtracting the PPDA OD_480-630_ from the PPDB OD_480-630_ values. The test-positive cut-off (OD 0.14) is shown by a horizontal line. Each symbol represents one dog sample. Test-positives are shown as solid symbols.

The comparative PPD ELISA (Figure 7) found generally higher responses to PPDA than to PPDB in the LRG population. The test cut-off was calculated as the mean OD [PPDB-PPDA] of the TB-free group plus three standard deviations, providing a cut-off value of 0.14. Using this threshold gave a 100% specificity and identified 27 test-positive HRG dogs (16.6%).

### 3.3. Serum IFN-γ ELISA & TNF-α ELISA Assays

No statistical difference was found between the quantities of either TNF-α or IFN-γ present in the peripheral circulation of the dogs comprising the HRG and LRG.

### 3.4. Haematology and Serum Biochemistry

Standard haematological and serum biochemical analysis was conducted on samples collected from half (*n* = 83) of the HRG dogs, selected at random. The only haematological abnormality identified frequently was an increase in the total leukocyte count (in 22 [26.5%] HRG dogs); in most cases this was due to the presence of a mature neutrophilia (in 18/22 of these HRG dogs), while band neutrophils were also present in a minority of cases (in 4/22 of these HRG dogs). One dog was found to have each of eosinophilia, monocytosis, and reticulocytosis.

Only five dogs of the 83 HRG dogs tested (6.0%) were found to have all serum biochemical tests within the reference interval (RI). The most frequently detected abnormality was an elevated serum urea, which was above RI in 44/83 dogs (53.0%), 3 of which also had elevated bile acid concentrations. The total calcium was low in 18/83 HRG dogs (21.7%), whilst alkaline phosphatase (ALP) activity was reduced in 25/83 (30.1%) HRG dogs but increased in 6/83 HRG dogs. More than a quarter (22/83 HRG dogs) were found to have elevated globulin levels; 20 of them were elevated enough to increase the total protein concentration of the serum to greater than RI with normal albumin concentrations. No association was found between these changes and the results of the other tests.

## 4. Discussion

Almost every species of mammal, including the domestic dog, is susceptible to infection with MTBC organisms, including *M. bovis* [2,3,4]. Canine disease due to *M. bovis* has been documented in a number of clinical case reports over many decades, with several reports demonstrating inter-species transmission, including to and from humans [1,2,37,38,39,40,41,42,43,44].

Two recent reviews analysed new and historical case records relating to all of the 583 identifiable cases of canine tuberculosis distributed worldwide [45,46]. These were all caused by one of three organisms: *M. tuberculosis* (55.2% of speciated isolates), *M. bovis* (35.0% of speciated isolates), and *M. microti* (0.9% of speciated isolates). The case distribution over time showed that the incidence of *M. bovis* tuberculosis in dogs has increased significantly again between the years 2000 and 2020, leading it to be classified as a re-emerging pathogen of global concern with twice the number of *M. bovis* cases diagnosed compared to *M. tuberculosis* cases in the last ten years [45,46]. Both of these studies highlighted the need for adjunctive diagnostic tests to support early treatment decisions, as current diagnostic testing for TB in the dog is so limited. There is a single study describing the use of an IGRA for the detection of *M. tuberculosis* in a group of 40 dogs living in close contact with sputum-positive pulmonary TB owners [16]. To date, no other tests have been systematically evaluated and found to be effective for the diagnosis of canine MTBC infections; intradermal TST for both *M. bovis* and *M. tuberculosis* is unreliable, as are complement fixation and haemagglutination methods [1,3,4].

In GB, *M. bovis* infection is endemic, with the highest prevalence in the counties comprising the High-Risk Area of England and High-TB Area of Wales, and lower but still significant prevalence within Intermediate or Edge Area counties. In these areas, species other than cattle may also be infected with *M. bovis*, and it was from within the Edge Area that we previously reported an outbreak of *M. bovis* disease in a pack of kennelled foxhounds [5,47,48]. The lack of reliable diagnostic assays meant that the prevalence of subclinical *M. bovis* infection within the group of exposed animals was challenging to determine, and potentially led to the outbreak lasting longer, and costing more to bring under control, than if such a test or tests had been available.

To begin to address these shortcomings, we have in this study applied commercially available tests produced for other species (Idexx for cattle, DPP VetTB for cervids) with an in-house serology test, and cellular cytokine release assays (IFN-γ and TNF-α) using commercial reagents and kits, for their ability to detect subclinical infections in dogs from both high- and low-expected prevalence populations. All of the tests chosen evaluate the host immune response rather than relying on direct pathogen identification or isolation. Previous studies have demonstrated that the major immunological consequences of MTBC infections are broadly conserved across host species [4,49]. Once mycobacteria are phagocytosed, macrophages and dendritic cells present antigens from lysed bacilli to CD4^+^ and CD8^+^ αβ as well as γδ T-cell subsets in the context of cell-surface MHC II molecules. The reciprocal activation between cells of these immunophenotypes, through the secretion of IFN-γ and TNF-α along with other cytokines, generates the classical cell-mediated response required for the control of an intracellular pathogen.

The first diagnostic test we assessed was an adapted IGRA, identifying animals with IFN-γ-producing antigen-specific T-cells. Similar to domestic cats, lions, and new world camelids, we found that it was necessary to isolate PBMC from heparinised blood samples prior to antigenic stimulation in order to reliably generate interpretable test data under these conditions [21,23,27,49,50]. This was due to the failure of cells within the whole blood of these species to respond significantly to either positive control or antigen stimulation, unlike the whole-blood responses observed in cattle, goats, and humans [10,24,25].

Examining the responses generated by the HRG dogs, we found that 77 (47.0%) showed a PPDB-biased response, indicating subclinical MTBC infection, compared to the LRG/TB-free groups, where only two animals (2.7%) showed this response (interestingly, these were the two dogs most in contact with the HRG hounds). These results suggest that this test method and interpretation have a high specificity of 97.3–100%, which is within the range of 90–100% consistently reported for IGRA assays in cattle, cats, and goats [10,24,25]. Of the 77 HRG dogs showing a PPDB-biased response, 62% simultaneously showed a significant response to ESAT-6/CFP-10, which is strongly suggestive of infection with an RD-1^+^ MTBC organism; this is slightly lower than the proportion reported for both cattle (~75% in the high-specificity IGRA, where both the PPD and peptide must be positive, and 82% for peptide alone as a diagnostic) and cats (80%, but too few cats for significance) [10,50,51]. This difference may reflect a lesser immunological significance of these antigens in canine infections and/or may be an artefact of the unknown incubation time from infection to blood sampling in the HRG dogs; had infections been allowed to continue to incubate, the number of dogs responding to these peptides may have increased.

A further five dogs in the HRG only responded to the peptide cocktail ESAT-6/CFP-10. With a lack of an antigen-specific PPDB response, it is questionable whether these animals are truly infected with *M. bovis* or not, since some NTM (notably *M. kansasii* and *M. leprae*, among others), encode homologs of these proteins, and as the HRG in this study comprised working foxhounds, there was significant potential for them to be exposed to/infected with confounding NTM. However, infected cattle are known to respond to ESAT6/CFP-10 in the absence of a positive PPD response, and in a setting of confirmed *M. bovis* infection such as the HRG dogs, a positive response to these peptides suggests an individual at a high risk of being infected. Furthermore, no animals in the LRG/TB-free groups demonstrated ESAT-6/CFP-10 responses, despite many having a similar opportunity for NTM sensitisation as the HRG. Thus, whilst the potential for a confounding influence of NTM is important for interpreting individual animal responses, at a group level the data presented here would suggest that a response to ESAT-6/CFP-10, even alone, is sufficient to indicate a high risk of an *M. bovis* challenge.

In our previous assessment of test responses, individual dogs with a significant response to any test antigen were considered test-positive [5]; this included three dogs that responded to PPDA alone. An analysis of the LRG tests found that this response occurred in 12 of the 74 dogs (16.2%), suggesting that this should not be seen as indicative of being at risk of *M. bovis* infection. Therefore, the definition of IGRA-positive animals in the context of subclinical *M. bovis* infections can be redefined to consider only individuals who show a statistically significant response above the media control to PPDB that is greater than the PPDA response, and/or those who respond significantly to the ESAT-6/CFP-10 peptide cocktail.

The diagnostic sensitivity of assessing antigen-specific TNF-α responses has only recently been tested clinically on relatively small populations of *M. tuberculosis*-infected people, with inconclusive results; however, it shows promise for discriminating between active and latent disease [52,53]. The approach we used, combining two ELISA cytokine measurements (IFN-γ and TNF-α) on the same stimulated cell supernatants, identified additional test-positive individuals, suggesting that a dual-cytokine test approach may have a higher diagnostic sensitivity without the need to take any additional blood from the animals being tested. However, going forward, there would need to be a modification of the positive mitogen control to include ConA, since PMA/Ca alone did not stimulate canine TNF-α release.

The detection of specific antibody responses has been useful in the development of new diagnostic tests for mycobacterial infections, particularly for species where the repeat capture of the same individual at a fixed time point is difficult, and therefore precludes the use of TST [32,54,55], or where reagents for IFN-γ are not available, and/or an IFN-γ test might be viewed as disproportionate given the scale of problem. We therefore examined the antibody responses of dogs using a combination of serological tests already in use for other species.

As expected, the total number of test-positive animals identified by the serological assays was significantly lower than for the PBMC IGRA, with 16.5%, 7.4%, and 6.7% for the PPD ELISA, Idexx ELISA, and DPP VetTB tests, respectively, compared to 52% IGRA-positive results in the HRG dogs. The comparative PPD and Idexx *M. bovis* Ab ELISAs and the DPP VetTB assay all identified small numbers of animals not found to be positive by IGRA. Overall, there was generally weak agreement between the results of cellular and serological tests. However, the simplicity of the antibody tests compared with the IGRA test does provide relative speed and a lower test cost compared to the IGRA, which could facilitate an initial blood test screening of large numbers of animals in confirmed breakdown situations, and/or provide a serological option where blood sampling and transport with PBMC remaining viable for an IGRA may not be possible.

Of the three antibody tests, the comparative PPD ELISA found a much higher number of test-positive dogs, namely 27 compared to 12 (Idexx) and 11 (DPP VetTB). Whether this is due to PPD providing the largest epitope repertoire of all assays tested, or whether the test cut-off is not optimal, remains to be explored. Many LRG/TB-free dogs had a PPDA-biased response resulting in a very low test cut-off (0.14) compared to the Idexx ELISA (0.3).

Evaluating all of the test responses for each individual dog, it became clear that a small minority of individuals showed much higher responses to the tests than the rest of the test-positive population on each assay. Further examination showed that this was consistently due to three individuals for the cell-based and cytokine assays, and a single individual for the antibody-based assays. The relevance of the magnitude of this test response is unclear. There is conflicting evidence in the scientific literature as to whether the concentration of cytokines in serum and/or diagnostic assays produced in vitro is proportionate to the severity or progression of clinical disease in vivo [56,57,58], whilst there is some evidence indicating that ESAT-6/CFP-10-specific IFN-γ responses correlate with disease severity in cattle and that the titre of detectable antibodies correlates with the occurrence of a lesioned phenotype [24,30]. This may be true for dogs; however, data in this study did not support this view, possibly because due to the subclinical nature of the HRG tested.

Haematological abnormalities were identified infrequently in the 83 dogs in the HRG that were tested. The high total leukocyte counts and mature and immature neutrophilia seen in a small number of dogs did not correlate with any of the mycobacteria-specific diagnostic tests; they probably reflect an unknown inflammatory stimulus in these dogs.

Serum biochemistry of the same dogs revealed multiple frequent abnormalities. The most frequent, elevated urea concentration, likely reflects the high-protein diet of the working hounds, as they were fed raw meat, rather than any underlying pathology. Half of the 22 dogs with hyperglobulinaemia were also found to be positive to at least one of the serology assays, indicating that in some cases, hyperglobulinaemia may have been related to the presence of antigen-specific antibodies. However, the overall specificity of hyperglobulinaemia with respect to the remainder of the tests was poor.

This study was unable to evaluate test sensitivity, as too few confirmed infections were identified—in total seven confirmed clinical cases of *M. bovis* infection occurred during the course of the kennel outbreak. Due to the severity of the disease, these affected animals were euthanized on welfare grounds before diagnostic blood samples could be taken. The diagnosis of *M. bovis* infection in these animals was confirmed by the mycobacterial culture of visibly lesioned tissues collected post mortem in each of these cases. The rate of false-negative test results (type II error) in this study is therefore also unknown.

## 5. Conclusions

In summary, this study demonstrates, for the first time, the utility of two cellular (IGRA and TNF-α) and three serological (DPP VetTB, Idexx, and comparative PPD ELISA) assays for detecting sub-clinical *M. bovis* infections of dogs. Whilst the data suggest a high test specificity for all assays evaluated, further work is needed to robustly validate the specificity of the tests and investigate the test sensitivity using sufficient numbers of dogs of confirmed infection status.

## Figures and Tables

**Table 1:** Origin of blood and peripheral blood mononuclear cell (PBMC) samples used in this study categorized by the risk of *Mycobacterium* (*M. bovis*) infection. * Serum was unavailable for one foxhound; HfSA—Hospital for Small Animals. ** Samples acquired from dogs resident in Scotland, which has held Officially TB-Free (OTF) Status since 2009.

Risk Status for *M. bovis* Infection	Source	Total Number	Number of Serum Samples	Number of PBMC Samples
High risk	Kennel foxhounds where an *M. bovis* outbreak occurred	164	163 *	164
TB-free **	Canine patients at the HfSA, University of Edinburgh	45	45	0
TB-free **	Blood-donor dogs at the HfSA, University of Edinburgh	24	0	24
TB-free **	Canine geriatric health screen patients, HfSA University of Edinburgh	8	0	8
Low risk	Pet dogs in contact with a household (animal) case of mycobacterial disease	10	0	10
Low risk	Pet dogs of kennel staff where a previous *M. bovis* outbreak occurred	32	0	32

## Data Availability

The original contributions presented in the study are included in the article; further inquiries can be directed to the corresponding author.
